# Immune modulation through targeting PD-1/PDL-1 signaling pathway for the treatment of osteosarcoma lung metastastasis

**DOI:** 10.1186/2051-1426-3-S2-P218

**Published:** 2015-11-04

**Authors:** Pooja M Dhupkar, Ling Yu, Nancy Gordon, Eugenie S Kleinerman

**Affiliations:** 1 University of Texas MD Anderson Cancer Center, Sugar Land, TX, USA; 2 University of Texas M.D. Anderson Cancer Center, Houston, TX, USA

## 

Osteosarcoma (OS) is a primary bone malignancy, commonly culminating into aggressive pulmonary metastasis. Despite chemotherapy advances, the 5-year survival of pulmonary metastatic OS remains 25-30%. Immunotherapy is one of the promising novel approaches to target minimal residual and relapsed disease. Previously, we have demonstrated that Liposomal muramyl tripeptipe phosphatidyl ethanolamine (L-MTP-PE), through a mechanism that involves macrophage activation, reduced the metastatic burden in animal models and caused an 8% improvement in the overall survival of patients with OS metastatic disease.

The goal of this study is to determine if PD-1-PDL-1 immunosuppressive signaling pathway can be targeted using PD-1 checkpoint inhibitors against OS lung metastasis. Upregulation of PDL-1 on tumor cells and its enhanced interaction with PD-1 immune-inhibitory receptor is one of the acquired mechanisms of tumor cells to decrease immune-therapeutic efficacy. Anti-PD-1 and anti-PDL-1 antibodies have exhibited therapeutic benefit in melanoma, renal cell carcinoma and non-small cell lung carcinoma. However, the effect of anti-PD-1 therapy and the role of PD-1-PDL-1 signaling in *metastatic* OS lung disease have not been investigated. We hypothesize that the disruption of the PD-1-PDL-1 signaling pathway using anti-PD-1 antibody is effective against OS lung metastasis and will improve the overall survival. Our data confirms surface and total PD-L1 expression in a panel of human OS cell lines by flow cytometry and Western blot analyses respectively. Furthermore, immunohistochemistry staining of primary and metastatic lung tumor samples from patients demonstrated membranous and cytoplasmic PDL-1 expression providing evidence that PDL-1 might be responsible to the limited OS response to immunotherapy. In addition, using a human OS mouse model we demonstrated therapeutic effect of anti-PD-1 therapy as evidenced by a decrease in the number of micro and macrometastases. Immunohistochemistry staining of lung tissues from mice treated with anti-PD-1 therapy demonstrated enhanced macrophage infiltration when compared with the control group. However, there was no significant increase in NK cell infiltration. We further demonstrated a decrease in PDL-1, p-ER1/2 and p-Stat-3 expression by Western blot analyses of lung tumors from mice receiving anti-PD-1 treatment. We conclude that anti-PD-1 therapeutic efficacy may be mediated by the enhanced macrophage infiltration in osteosarcoma pulmonary metastatic tumors. Targeting PD-1/PD-L1 axis might be a promising approach for the treatment of OS lung metastasis.

